# Communities of practice in Alberta Health Services: advancing a learning organisation

**DOI:** 10.1186/s12961-020-00603-y

**Published:** 2020-08-03

**Authors:** Anna M. Auer, Patricia Hanson, Barbara Brady-Fryer, Julie Alati-it, Allison L. Johnson

**Affiliations:** 1 Alberta Health Services, Knowledge Management Department, Provincial Clinical Excellence, Seventh Street Plaza, 10030 – 107 St, Edmonton, AB T5J 3E4 Canada; 2 Alberta Health Services, Knowledge Management Department, Provincial Clinical Excellence, Centre 15 Building, 100, 1509 Centre St. SW, Calgary, AB T2G 2E6 Canada

**Keywords:** Alberta Health Services, Communities of practice, Delivery of healthcare, Health region, Canada, Health organisation, Knowledge management, Knowledge sharing, Organisational change, Systems integration

## Abstract

**Background:**

In 2009, Alberta Health Services (AHS) became Canada’s first and largest fully integrated healthcare system, involving the amalgamation of nine regional health authorities and three provincial services. Within AHS, communities of practice (CoPs) meet regularly to learn from one another and to find ways to improve service quality. This qualitative study examined CoPs as an applied practice of a learning organisation along with their potential influence in a healthcare system by exploring the perspectives of CoP participants.

**Methods:**

A collective case study method was used to enable the examination of a cross-section of cases in the study organisation. Semi-structured interviews were conducted with 31 participants representing 28 distinct CoPs. Using Senge’s framework of a learning organisation, CoP influences associated with team learning and organisational change were explored.

**Results:**

CoPs in AHS were described as diverse in practice domains, focus, membership boundaries, attendance and sphere of influence. Using small-scale resource investments, CoPs provided members with opportunities for meaningful interactions, the capacity to build information pathways, and enhanced abilities to address needs at the point of care and service delivery. Overall, CoPs delivered a sophisticated array of engagement and knowledge-sharing activities perceived as supportive of organisational change, systems thinking, and the team learning practice critical to a learning organisation.

**Conclusion:**

CoPs enable the diverse wealth of knowledge embedded in people, local conditions and special circumstances to flow from practice domain groups to programme and service areas, and into the larger system where it can effect organisational change. This research highlights the potential of CoPs to influence practice and broad-scale change more directly than previously understood or reported in the literature. As such, this study suggests that CoPs have the potential to influence and advance widespread systems change in Canadian healthcare.

## Background

Canada’s publicly funded healthcare system affords each citizen or eligible resident reasonable access to medically necessary services. Federal criteria are set out to ensure that all eligible residents receive access without direct charges at point of service. Under Canadian law, the nation’s 13 provinces and territories are individually responsible for healthcare delivery [1], including the management, organisation and provision of healthcare services for their residents.

Until recently, each of Canada’s provinces and territories typically provided healthcare through province-based networks of regional health authorities (RHAs). However, in 2008, the Government of Alberta’s Ministry of Health took unprecedented legislative action to mandate the amalgamation of its RHAs into a single governance and management authority, creating Alberta Health Services (AHS) [2]. This amalgamation established Canada’s first fully integrated health service entity, the study organisation for this research. This service integration aligns with Canada’s rising imperative to provide better healthcare at lower cost [3, 4].

Operating under one corporate umbrella, AHS serves 4.3 million residents geographically distributed across approximately 660,000 km^2^. These services are provided by 114,400-plus employees located at more than 850 sites throughout the province, including hospitals, tertiary/quaternary referral centres, continuing care facilities, cancer care centres, mental health facilities, and community health clinics [2]. In addition, AHS provides specialty services for parts of Saskatchewan, British Columbia and the Northwest Territories [2].

In Alberta, the shift from RHAs to a single entity for healthcare provision was projected to improve patient experience and population health while decreasing overall costs. In part, this projection was centred on reducing practice variation and redundancies in clinical and corporate services across the province. The topic of healthcare integration has produced its own body of literature, which indicates that these results depend on three high-level approaches, namely a systems approach recognising the complex interrelations of people, process and technology [5]; an inter-professional commitment to efficient information transfer serving a strong patient-first focus [6]; and outcome-focused thinking [7]. These approaches are reflected in the study organisation’s Foundational Strategies [8] and conceptually linked with being a learning organisation.

The concept of the learning organisation was first introduced over three decades ago to bolster success in organisations facing challenging and complex issues by leveraging an internal learning framework [9, 10]. According to social systems scientist Peter Senge, the following five disciplines characterise learning organisations [10]:

- Systems thinking – involves understanding that discrete elements of an organisation are in some way connected to form patterns and that each element affects another.
- Personal mastery – depicts the ability of individuals to achieve what truly matters to them. In the workplace, this is a reciprocal commitment between individual employees and their employer.
- Mental models – represent assumptions that influence both our individual and organisational views of what is possible and not possible.
- Building shared vision – refers to a genuine commitment to act together, including creating and achieving goals together.
- Team learning – supports shared insights that make organisational goals attainable.

Across Senge’s five disciplines, team learning is singled out as being central to a learning organisation and most likely to benefit from regular practice [10]. Communities of practice (CoPs) exemplify team learning. Contemporary authors Wenger-Trayner have defined a CoP as “*a group of people who share a concern or a passion for something they do, and learn how to do it better as they interact regularly*” [11]. Senge notes that team learning is the power that allows CoP members to construct outcomes that they truly want [10] and to make a difference in people’s lives [12, 13], paving the way for the strong collaboration necessary to build a learning organisation [14] on a large scale. Similarly, the United Kingdom’s National Health Service has recognised CoPs as enablers of large-scale public service change, by providing effective and gratifying ways for staff to capture and pull diverse forms of knowledge together [12].

Early conceptions of CoPs were associated with Swiss-American researcher Etienne Wenger [15], considered by many to be the originator of the concept. Wenger built on the work of his contemporaries, with social anthropologist Jean Lave [16], to examine the connections between social processes, learning and apprenticeship. Wenger et al. [17] identified CoPs as promising tools for organisational management of knowledge and useful mechanisms for team learning in complex work environmentsr.

Systematic reviews and clinical practice studies [18] have highlighted significant opportunities for CoPs to substantially influence healthcare. CoPs have come to be recognised as vehicles for important healthcare system advancements such as increasing integration between primary and tertiary care to reduce unnecessary referrals [19], promoting change adoption to improve the care of seniors [20], and improving the uptake of care practices and new practitioner mentoring in public health [21]. The study organisation recognises CoPs as learning and capability assets in its employee development resources, a link also seen in best-practice organisations studied by the American Productivity and Quality Center [22].

Critics of Senge [23, 24] have noted that the theorist stops short of providing real-world instruction on implementing practices to promote functioning as a learning organisation. These criticisms reflect a divide between the theory of a learning organisation viewed as a “*tool, a lever, and a philosophy*” [14] and applied knowledge about CoPs functioning in a learning organisation. The use of CoPs and the five disciplines of a learning organisation are reflected in the published literature as being complementary in both theory and practice. However, at times, the five disciplines or practices are absent from discussions linking Senge’s learning organisation and CoPs [25].

### Purpose of the study

The aim of this study was to address the knowledge gap about CoPs as an applied practice of learning organisations by exploring the perceptions and experiences of CoP participants within a health services organisation. The study examined the following research questions in the context of the study organisation:

- How are CoPs structured and how do they function?
- Do CoPs influence healthcare practice and patient care?
- Does team learning in CoPs influence change within a large healthcare organisation?

## Methods

A descriptive collective case study method [26, 27] was used to examine the contextual experiences of CoP members in the study organisation. For the purposes of this study, a case was defined as a CoP. Data obtained for each case was comprised of study participant perspectives (i.e. CoP member, facilitator and/or sponsor) captured during interviews. This qualitative method [28] was chosen to ensure that the detailed nuances of CoP participant perceptions and experiences were examined in sufficient depth [29]. The use of the collective case study method [28, 29] enabled the examination of a cross-section of cases in the study organisation. The five disciplines in Senge’s framework, viewed from a practice perspective, were used in data analysis to interpret and map CoP influences associated with team learning and organisational change [29, 30].

### Study sample

Participants were primarily recruited through the Community of Practice Facilitators’ Network (CoPFN), an established CoP operated by the study organisation’s Knowledge Management department. With a distribution list of over 150 contacts, this Network’s membership is open to all CoP facilitators within the organisation. CoPFN contacts were invited by e-mail to take part in the study, generating an initial response of 25 contacts expressing interest in participating.

A criteria-based pre-screening process was used to ensure that potential study participants had the required experience to share relevant information and insights about CoPs. Criteria were derived from healthcare-based CoP literature and potential study participants were asked to consider which criteria applied to their respective CoPs. Those who identified with at least two CoP criteria were included in the study (Additional file 1).

Using a snowball sampling strategy, eligible research participants were invited to identify additional participants [31] who were also screened using the same criteria-based method. The research team sought to identify unique CoPs in the screening process; however, initial CoP names provided by participants varied, resulting in 2 study participants for 3 of 28 unique CoPs. One eligible participant withdrew from the study prior to an interview being conducted, having left employment with the organisation. Thirty-one participants representing 28 CoPs were enrolled in the study. The study sample included 20 CoP facilitators (who coordinate and facilitate meetings), 8 CoP members (who attend meetings of interest); and 3 CoP sponsors (who formally support CoP interests and participation).

The background of study participants was also varied and included nurses, allied health professionals, technicians, analysts, administrative staff, and managers from frontline through to the executive leadership level. Although physicians are members of some of the CoPs contacted during recruitment, no physicians volunteered for the study.

### Data collection

Semi-structured interviews were conducted over a 10-week period spanning from April through to June of 2017. All interviews were scheduled at a time convenient to participants and took place virtually using Microsoft Skype for Business, a communication platform that also supported the audio recording of interviews. Study participants received a formal invitation for participation outlining the research process, how the data would be used, stored, and reported and conditions associated with consent. Verbal consent was witnessed and documented by two members of the research team. Following the establishment of consent, interviews were initiated based on questions provided to study participants in advance of the interview. The average length of interviews was 1 hour.

Participant interviews involved two research team members, with the exception of one interview for which consent was verified by audio recording and a secondary review by the research team. One research team member was responsible for leading the interview while the second team member took notes of significant remarks and provided technical support for the Skype audio recordings. In the event that an interviewee made a remark or comment that went unnoticed by the lead team member, the second team member assisted with prompting the interviewee for additional information or clarification.

Verbatim transcriptions of each recorded interview were prepared and reviewed by the two team members involved for accuracy and completeness. Any lengthy or difficult to discern transcriptions were reviewed by a third member of the research team. Data collected for each of 3 cases yielding 2 participants each was aggregated for analysis pertaining to CoP structure and function. Audio recordings and the transcriptions were stored in digital format using Microsoft SharePoint, on a collaborative web-based site restricted to the research team.

### Data analysis

NVivo 11 data management software was used for data analysis. Two researchers initiated analysis using template coding [32] based on the study’s primary interview questions and prompts. Emergent topics in the data were then coded and refined throughout the analysis process. Thematic saturation and distinct insights emerged after 23 transcripts were coded and analysed. Initial templates were structured using primary interview questions and prompts, and matured through the use of theory-driven immersion crystallisation, a process of intensive reading and contemplation of the data (immersion) that allows for patterns, topics and themes to emerge (crystallisation) [32].

Transcripts were independently reviewed and coded by two team members on separate databases, then jointly compared and reconciled. Separate NVivo databases were merged following iterative reconciliations and coding revisions. CoP attributes described by interviewees were coded and categorised and subsequently cross-referenced by the research team to reach common agreement on discrete coding definitions and categories. Throughout the data analysis process, emerging themes and patterns were reviewed and discussed to ensure alternative interpretations were explored. Cross-verifications were performed at regular intervals to address potential interpretive bias and to ensure credibility and confidence in the results [33].

The method of analysis was iteratively sequenced. Initially, individual CoP case descriptions, perceptions and experiences were examined to consider the distinct attributes of each case and to identify information sharing and exchange patterns across the organisation. The attributes of individual cases were then examined to identify and compare attributes and thematic patterns across cases. Next, the influence of CoPs, as constituents of a learning organisation and team learning as a learning organisation practice [10] were examined.

Validation of findings and interpretations was undertaken by way of three-member checking review sessions [34]. Three sessions were constructed to encourage research participants to engage in open discussion and reflection, while also providing feedback and clarifications. At the end of each session, additional comments were invited by e-mail. Feedback gathered from these three sessions was summarised and sent out to all study participants along with another invitation to provide feedback through Skype-based discussions or e-mail. One-on-one validation-focused Skype conversations were conducted with those participants that were unable to attend a member checking review session, yielding an overall uptake of 93% of study participants in the validation process. Feedback from the validation process was incorporated as data within the study’s main findings.

## Results

### How are CoPs structured and how do they function?

The diversity of CoPs emerged as case-specific attributes were defined, categorised and compared. An examination of CoP attributes identified the following structures and functions (Additional file 2).

#### Practice domain

Practice domains of the CoP members represented a broad cross-section of the following areas of expertise within the study organisation: quality improvement, employee engagement, project management, patient safety, patient relations and concerns, patient- and family-centred care and services, frontline management and supervision, organisation-wide support services, and data analysis.

#### Focus

An interview question invited participants to describe the focus of their CoP. The reported focus was also diverse and included supporting frontline research involvement and dissemination, developing patient resources, supporting development and advancement of professional competency standards, providing mentoring and professional development opportunities, creating information sharing networks, furthering project initiatives, establishing forums for community input, facilitating interprovincial communication, and managing case conferencing.

#### Membership boundaries

CoPs formed membership boundaries as they refined their focus. These boundaries were shaped and defined by fundamental considerations such as staff roles, practice domain and patient confidentiality. Membership boundaries varied significantly from being open to anyone within the study organisation and professional groups to being highly restricted based on staff roles and patient confidentiality issues (Table 1).

**Table 1:** Classification of Communities of Practice (CoPs) by membership boundaries

Membership boundary categories	CoPs in cohort (*n* = 28)	Basis of categories	Sample membership descriptions
Unrestricted	4	Members are part of the practice community based on subject-matter interest and/or expertise and may include non-AHS staff (i.e. not bound by role, geographic boundaries or confidentiality restrictions)	“*… open to anyone, we’re a very eclectic group … positions where people are directors, experts in change management, non-experts interested change management, and professional groups*.”
Restricted by domain and role	10	Membership is characterised by being practice and role centred	“*… open to those in our* [lead] *roles to establish a network for best practices*.”
Restricted by domain and geography	6	Membership associated with geographic boundaries (e.g. site, zone based) and role-based requirements	“*… open to all acute care, all direct service staff who have palliative care interactions with patients and families and aren’t specialized. It’s inter-professional and it’s specific to our site*.”
Restricted by role and confidentiality	8	For privacy considerations, membership is strictly bounded by staff roles (e.g. associated with sharing identifiable patient information) or practice-specific patient information	“*… open only to those in our* [particular] *role as we discuss patient* [related] *information*.”

Note: Alberta Health Services integration defined five geographic ‘zones’ to distinguish service clusters in the context of Alberta’s expansive land base

#### Meeting attendance and methods

Study participants reported average attendance at CoP meetings by estimate and these figures varied. The highest range for average attendance was reported as being 50–100 attendees and the lowest range reported was ≤10 attendees. Overall, 22 CoPs reported meeting virtually using Skype for Business, while 4 CoPs reported meeting by phone and 2 CoPs described meeting face-to-face.

#### Sphere of influence

Sphere of influence as a CoP attribute reflected both the geographical and functional spheres of influence of a CoP within the study organisation. Spheres of influence were broadly ranged – some CoPs were categorised as province, organisation or multi-zone wide, while others were categorised as being zone or site specific (Table 2).

**Table 2:** Classification of Communities of Practice (CoPs) by sphere of influence

Sphere of influence categories	Classification of CoPs in cohort (*n* = 28)	Basis of categories
Province wide	6	Focusing on issues important to AHS and involving partners external to AHS
Organisation wide	10	Influencing internal organisational functions impacting all of AHS
Multi-zone wide	4	Functioning across more than one defined geographic zone or service cluster
Zone wide	7	Functioning across one of five geographic zones or service clusters
Site specific	1	Functioning, focused and situated at a single service site

*AHS* Alberta Health Service

### Do CoPs influence healthcare practice and patient care?

CoPs do influence healthcare practice and patient care by the following means: providing meaningful interactions, building information pathways, and building capacity to address patient needs at the point of care and service delivery.

#### Meaningful interactions

Meaningful interactions were one of the most commonly described benefits of CoPs, independent of practice domains. They were viewed by participants as influencing practice and patient care by improving staff engagement and promoting a sense of capacity to create and improve upon things that make a difference. Study participants also described CoPs as providing a space for sharing ideas and a safe place to connect and learn.

Notably, participants often identified more than one positive influence of these meaningful interactions on practice and patient care. Meaningful interactions ameliorated geographic isolation, influenced better practice, and provided opportunities for focused team learning. Further, study participants identified that CoPs supported better practice by strengthening their professional voice and identity (Table 3).

**Table 3:** Meaningful interactions – themed influences on healthcare practice and patient care

Themes	Participant quotes	Role
Safe space to connect and learn	“*What goes on in the meeting is confidential and we’re safe to say whatever it is we want to say in there without people, you know, disrespecting each other or taking it outside*.” (Multi-zone wide CoP – 007)	Member
	“*The frontline staff wanted to continue to have a safe place where they could meet and share their practice stories and experiences after that initial training …*” (Zone-wide CoP – 024)	Facilitator
	“*So it’s a bit of a balance of having experts available to provide content but then also having a platform that’s safe for conversation and knowing that you can ask a question and, you know, get a good response without being criticized or judged or having somebody question your clinical skills or something like that*.” (Province-wide CoP – 031)	Member
Interpersonal connection	“*First year, focused a lot on forms of socializing as a team – things like how you view and understand your colleagues, shared experiences*.” (Multi-zone CoP – 017)	Facilitator
	“*We don’t go out to coffee together and we started on a virtual platform because we had to and we started to form personal and professional relationships*.” (Zone-wide CoP – 011)	Member
	*“… being rural and the only person around in your area … it can be very isolating, so you have that feeling like you have some kind of community*.” (Zone-wide CoP – 025)	Facilitator
Better practice	“*We’re meeting as a* [CoP] *team every week and we … discuss – how do we move our program forward, how do we do things differently, how do we make improvements, how do we change things?*” (Zone-wide CoP – 022)	Member
	“*It absolutely* [has] *changed a lot about the way I work. It’s really helped our communications and information sharing … we have a place to go to communicate …*” (Province-wide CoP – 018)	Facilitator
	“*CoPs help improve patient care and family care, so anything that helps staff support patients and families and improve care is a definite bonus*.” (Site-specific CoP – 002)	Facilitator
Professional voice and identity	“[CoP members*] have ownership and voice in setting direction*.” (Zone-wide CoP – 028)	Facilitator
	“[Our] *group contributed content and our opinions on how to develop* [resources] *and now it’s going to get shared back with the group as a province-wide* [resource/standard].” (Enterprise-wide CoP – 021)	Sponsor
	“*Now the whole team looks at competency profiles and works to achieve the goal collectively … and has shared ways for other sites to build on that*.” (Enterprise-wide CoP – 006)	Facilitator
Focused team learning	“*Getting people together … so much can be accomplished by getting like minds together – problem solving and sharing of information.*” (Zone-wide CoP – 022)	Facilitator
	“*When a site talks about how they struggled and how they overcame that struggle – and especially how that has led to success – that is invaluable*.” (Enterprise-wide CoP – 006)	Facilitator
	“*It’s so energizing to see* [CoP member] *excitement about what we’ve talked about or what [members] learned and how they’re going to implement it – it’s very satisfying*.” (Site-specific CoP – 002)	Facilitator

*CoPs* Communities of Practice

In summary, study participants identified multiple and diverse examples of meaningful interaction in CoPs adding value to healthcare practice and patient care.

#### Building information pathways

The second most-frequently and readily described influence on practice and patient care was found in the way that CoPs produced numerous information pathways to enhance information flow in the study organisation [35].

Study participants perceived these information pathways as transcending roles and organisational boundaries, expanding the potential for timely knowledge sharing in the provincially integrated study organisation.

#### Capacity to address patient needs at point of care and service delivery

The third CoP influence on practice and patient care highlighted by participants was the capacity to address staff and patient care issues at the point of care and service delivery. Change that was ordinarily implemented in a traditional top-down manner was accomplished at the point of care, where CoPs could optimise the collective use of frontline knowledge and experience with less lag time between problem identification and solution generation (Table 4).

**Table 4:** Influences on healthcare practice and patient care - information pathways and point of care capacity

Influences	Participant quotes	Role
Information pathways	“*On my team,* [the CoP] *does allow me to bring forward a perspective from the* [geographic] *zones. So I am around a lot of provincial teams in other departments as well so I can then bring their* [CoP] *voice to other tables, and to my team*.” (Enterprise-wide CoP – 021)	Sponsor
	“*The team knows that I and* [others] *are linked to other CoPs and they’re now asking us to bring the information from those CoPs back to ours to help them expand their information*.” (Zone-wide CoP – 026)	Facilitator
	“*Only through that dialogue and through networking could we create a situation where it would it become part and parcel of the business that we do*.” (Enterprise-wide CoP – 013)	Member
Point of care capacity	“*Solutions are coming from the grassroots that no one person or site could have come up with. Shows the power of collective sharing of the good and the bad*.” (Enterprise-wide CoP – 006)	Facilitator
	“*I might see a nurse who’s on the floor who is a* [CoP] *member and maybe we have a difficult case, I can touch base and say ‘hey, do you know anything about this patient’, and engage in conversation*.” (Site-specific CoP – 002)	Facilitator
	“*We might in turn share how a particular strategy worked for that* [patient] *file that* [members] *might try, you know, that might influence someone else’s approach to a similar file …*” (Zone-wide CoP – 011)	Member

*CoPs* Communities of Practice

CoPs and their information pathways at the point of care and service delivery enabled participants to use collective means to respond more accurately and rapidly to situations as they occurred. Over time, the enhanced capacity to respond nimbly had a discernible influence on change adoption in the study organisation.

### Does team learning in CoPs influence change within a healthcare organisation?

Study participants identified and described CoP membership actions and successes that they viewed as team learning successes producing benefits for the study organisation. The organisational benefits identified were promoting innovation, supporting employee retention, advancing process and practice standardisation, nurturing psychological health, expanding research participation and dissemination, contributing to talent management and professional development, and improving workload measurement.

Study participants viewed CoP member actions and successes as advancing change in the study organisation. According to participants, the potential value and role of CoPs in influencing organisational change is not yet widely understood within the study organisation. CoP member actions that resulted in scheduling improvements, cost savings, reduced turnover, and managing risks unnoticed or unknown to the organisation were welcomed; however, these successes were not consistently credited to CoPs (Table 5).

**Table 5:** Individual Community of Practice (CoP) membership successes collectively influencing systems change in Alberta Health Service

Individual CoP membership successes →	Collective influence of CoPs for systems change
• Establishing rules and accountabilities for data among data stewards	Innovation/solutions
• Scheduling changes and realised cost savings through collaboration in a provincial clinical service	
• Developing a single-source glossary for a specialty area, validated by experts active in the field	
• Enabling reduced turnover by connecting remote rural supervisors	Employee retention
• Creating a virtual space where geographically isolated colleagues connect	
• Instituting competency development for mandated care	Process and practice standardisation
• Adopting organisation-wide standardisation of best practices and processes	
• Managing risks of issues previously unnoticed/unknown to the organisation	Risk management
• Establishing a safety valve in geographically dispersed area with challenging case file support, challenging workloads and typically high turnover	Psychological health and safety in high-pressure settings
• Finding high-quality evidence for decision-making	Talent management and professional development
• Advancing change management and project management	
• Evolving subspecialty care practices	
• Mentoring new employees	
• Growing networks	
• Creating and adopting a case load intensity tool	Workload measurement

Often, CoPs were deemed as a time-consuming add-on to main responsibilities and/or a passion project in the context of a work environment that did not recognise their value. Research participants routinely described activities associated with preparing, facilitating and supporting CoP membership activities as being undertaken “*off the side of my desk*”. The meaning of this commonly used phrase was associated with CoP activities being viewed by supervisors as a non-designated work priority.

At member checking sessions, participant statements consistently centred on the need for all levels of AHS to understand the value and importance of CoPs at the point of care and service delivery, and the value of CoPs in contributing to advancing organisational change. Feedback statements summarised from member checking sessions and validated by participants were as follows: CoPs enable connections for those in similar roles working in geographic isolation, CoPs create pathways for information sharing, CoP benefits surpass their relatively modest resource requirements, CoPs spark innovation and problem solving, CoPs spot issues before the organisation does, and CoPs fast-track change adoption.

These statements capture participant perspectives on the value and influence of CoPs in AHS and their value in advancing the organisation’s transition from multiple regional authorities to one integrated, province-wide health system.

## Discussion

Achieving systems change through organisational level impacts is one of the most daunting challenges facing Canada’s healthcare system. The study organisation represents a notable example of systems change in the broadest sense, in that it is the first health system in Canada to integrate multiple regional health authorities and services under a single corporate umbrella [2].

The integration process exposed practice and service delivery gaps, duplications between sites, and policy and practice variations across departments and geographic zones. At the same time, this ongoing change process brought about many opportunities for streamlining and unifying the study organisation as one large province-wide enterprise. Findings of this research highlight the opportunities that emerged from ground-level CoP functions and activities, enabling the study organisation to gain momentum in the practice of team learning and thus advance as a learning organisation [10].

This study provides a limited but crucial view into the structure, function and experiential impacts of CoPs in the earliest years of establishing a large integrated healthcare organisation. As the study organisation matures, CoPs will continue to act as a mechanism to illuminate real-time issues for staff and patients adapting to widespread change at the point of care.

### Team learning and systems thinking

In 2010, Wenger called CoPs “*the smallest social unit that has the characteristics of a social learning system*” [36]. CoPs provide individuals and teams with the opportunities and platforms to share knowledge, innovate and pursue personal mastery over the challenges they encounter [36]. In this way, CoPs in the study organisation leverage Senge’s practice of team learning to benefit not only the membership but also the organisation on a broader scale.

In CoPs rooted in a learning organisation, ideas and innovations can be tested and either fail or succeed quickly, and the resulting information output can be transmitted throughout the larger system, enabling new connections and fostering an incremental build towards more substantial change. Systems thinking, another key practice in Senge’s learning organisation construct, develops as CoP members see how their actions disrupt standard operational patterns to create change in the system at large [10].

Critics of Senge’s conceptualisation of the learning organisation view it as an aspirational ideology rather than a useful action plan for guiding change [24, 37]. These views are countered by examining and detailing the alignment of CoP impacts with Senge’s practices in the study organisation. To the extent that CoPs influence how the membership learns and adapts to major change, CoPs can be considered as an applied practice of learning organisations faced with complex changes.

In a resource-restricted service delivery environment, service integration and its impacts in the study organisation inspired a willingness among staff to start up and nurture CoPs. At the same time there is considerable tension associated with releasing staff from primary roles to contribute to an undefined outcome, even in an environment where engagement is always encouraged.

### A shifting stance regarding CoP structure

Over time, Wenger et al. [38] have transitioned from the view that authentic CoPs are self-organising and cannot be created intentionally, to a view that CoPs can be cultivated deliberately. This revised stance recognises that CoPs can be designed and managed with the intention to improve patient care, services and organisational efficiencies [39]. While not specifically questioned on the point, study participants described both CoPs that had been generated from the top-down and were nurtured by sponsors as well as CoPs that originated from the bottom-up and then explicitly sought sponsorship to continue.

Within the confines of the study organisation’s context, this study supports the perspective that deliberately constructed CoPs can confer organisational advantages equal to that of their self-organised counterparts. Consistent with the evolution of CoP literature, emphasis from study participants was less on how CoPs were constructed and more on their value to membership and the organisation [40, 41].

### Building information pathways and boundary spanning

When describing how CoPs in the study organisation are structured and function, membership boundaries were variously defined by subject-matter expertise, practice and role, role-based requirements specific to geographic boundaries, and privacy considerations. In essence, these boundaries represent organisational subcultures where members could expect a shared language and a level of trust conferred by the presence of peers. However, for CoPs to influence the organisation beyond their local boundaries, boundary spanning was required.

The function of informational boundary spanning was originally described in the literature as extending from the inside to the outside of the organisation [42]. More recently, discussion of boundary spanning includes communication between and across departments or disciplines, and even global interchange [43–45]. Study participants attested to both internal and external boundary spanning, as a logical consequence of boundaries extending beyond the study organisation to include regulatory bodies, non-governmental organisations and employees of the provincial government.

Informational boundary spanning is powerful because of its informal nature, low cost, efficiency and nimbleness. A demonstrably effective communication method, boundary spanning takes place between members of CoPs who have been identified by their peers as model practitioners and technically competent sources of new information [44]. When the study organisation became a single entity encompassing an entire province, the demands for information exchange far exceeded the capacity of formal channels filtered through continuously shifting reporting structures. CoPs were able to improve information flow and transmission for their membership, a phenomenon that was remarked upon by many study participants.

Descriptions of boundary-spanning within the study cohort were positively framed and voluntarily assumed. Boundary-spanning behaviours were adopted exclusive of any ‘formal requirements’ linked to professional roles [46]. This suggests an organisational culture well-disposed to change-ready leadership practices [47]. Moreover, several CoPs reported receptive and constructive leadership responses to revealed gaps in patient care or service delivery. The findings of this study indicate that the overall culture of the study organisation facilitated boundary-spanning behaviour; however, outcomes and findings are likely to vary based on characteristics such as the entrenchment of social privilege and role legitimacy in the culture of study [48].

### Diverse practice domains and legitimate peripheral participation

In the interview cohort, facilitators reported being surprised to learn that sparse verbal participation by members did not equal a lack of interest or poor engagement. In one memorable narrative, a facilitator considered de-adoption for a CoP with a small core group of vocal participants, only to learn that the other members highly valued their experience, with some attending expressly to listen and learn. This phenomenon was described in Lave’s early work on CoPs as “*legitimate peripheral participation*” [16]. Among the CoP participants interviewed, some attended to test out new skills, some to collaborate, some to act as resources, and some to act as boundary spanners; engaging in group dialogue was not every participant’s aim. The amount and frequency of dialogue was often used by facilitators as an informal marker of a CoP’s success and the degree to which members valued the CoP. However, facilitators found that asking members directly about the perceived value of a CoP resulted in a more accurate assessment of its meaning and importance.

This is consistent with management literature in that CoP members have been said to engage in different ways as individuals and as subgroups [43]. There are many ways for members to contribute and learn that may go unnoticed such as gathering perspectives and strategies from a sector unfamiliar to them. The diverse practice domains observed in the study cohort made it possible for employees to explore more than one domain when seeking meaningful interaction by their own standards.

Through their voluntary nature, CoPs inspire passion projects close to the point of care and service delivery. Simply by attending a CoP meeting, participants are attending to what matters to them at a given time. Consequently, their perception of the value of the CoP to them and their work should not be judged by how verbally prolific they are.

### Micro-level changes influence and advance organisational change

By expanding analysis from the micro-level of examining what CoPs in the study accomplished as singular cases or entities to the macro-scale, where CoPs exercised collective influence on the organisation and its integration journey, contributions to organisational change can be seen. As summarised in Table 4, viewed in aggregate, CoPs in the interview cohort had a clear influence on the study organisation. While many of the member actions described as successes by interview participants had obvious linkage to person-centred care principles, collective influence was predominantly discernible in work force impacts.

CoPs in the study organisation are essentially constituents of the workforce supporting engagement, creating cultures in which members can feel safe, healthy and valued, and enabling each group to reach its full potential in the learning organisation. The human-centred nature of health service delivery firmly links employee enrichment to patient care through the positive effects of workforce engagement on patient and family experience. This is the operational, frontline link that makes it possible for CoPs to serve the needs and goals of the membership without forgoing those of the larger organisation.

### Modest investments and risk management rewards

One of the most prominent findings in terms of cost-benefit potential lies in the unique ability of CoPs to spot issues of risk at the ground level before they gain visibility within the hierarchy of an organisation. Provincial health services integration means that risks also have bearing on a provincial scale, escalating the prospective damage of negative outcomes. Because CoPs often operate at or close to the point of care and service delivery, risks can be identified and solutions put in place before a negative consequence is realised. Corrective and incremental actions, if needed, can take place where they will create the fewest adverse disruptions to the complex healthcare system.

For example, membership actions in a single CoP interviewed in the study cohort highlighted issues of potential patient risk and resulted in a robust mitigation response from senior leaders. While the diligent work of members and not the CoP itself are responsible for the absence of adverse events, mitigation activities have reduced the risk potential significantly. The broad sphere of influence established by CoPs in the study organisation confers dispersion and distribution of risk throughout a complex system where resistance to change would otherwise create barriers to integration.

### Strengths and limitations

This study used a theory-driven approach, with methods drawn from qualitative inquiry and informed by a cross-section of descriptive literature on learning organisations, large-scale change and CoPs. Examining and understanding how CoPs influence healthcare practice, patient care, organisational change and, ultimately, the healthcare system adds to the limited body of knowledge about CoPs in publicly funded healthcare systems.

In addition, the research team had varying knowledge levels and exposure to CoPs, both within the Knowledge Management department and beyond, which presented as both a strength and a limitation. Potential research bias was mitigated in multiple ways, including triangulation, uniformity and reproducibility of collection methods, independent and group interpretation and analysis, reviewing available CoP documents, and member checking sessions.

Findings are limited to this research cohort and cannot be assumed to be representative of all AHS CoPs. Potential study participants may have been constrained from participation by an inability to find time to respond from within their work environment, which may have reduced representation from CoPs operating in clinical settings. Most employees within the study organisation who have contact with CoPs do so as members, sponsors, or facilitators or sometimes as a combination of those roles. As a result, their view of the influence of CoPs in AHS was likely limited to some degree by the nature and scope of the respective CoP role of each study participant.

### Implications

A predominant view expressed by interview participants was disappointment that the potential of CoPs to make significant contributions is not well understood across the organisation. Participants also expressed being perplexed by this, given that, from their perspective, the benefits of CoPs well surpass their requirement for relatively modest resource requirements.

This was an unexpected finding as the CoPFN, which provided the initial distribution list for study recruitment, indicates broad support for CoPs in the study organisation. This finding has implications that will inform the Department of Knowledge Management’s strategic, operational and communications planning, given its role to support the development of AHS as a learning organisation. Since participants most keenly experience support or the lack thereof within their own line of reporting, this finding also has implications for facilitators and sponsors in defining expectations and roles associated with establishing and maintaining CoPs.

Other implications arising from this research centre on the potential of CoPs to facilitate valuable change and continuous learning in learning organisations. Though the concurrent existence of CoPs and learning organisations has been previously articulated in systems change literature, our findings indicate that the presence of CoPs, whether spontaneous, cultivated or some combination of the two, can foster visible and valuable change in learning organisations through incremental actions.

Within the complex environment of the study organisation, entering its second decade as an integrated provincial healthcare system, development and maturation as a learning organisation is ongoing. As the value of CoPs within the organisation becomes more widely known, this study, along with new studies exploring CoPs within a learning organisation, will help inform future actions to promote team learning and systems thinking and to record large-scale changes that develop as a consequence.

Future research is needed to advance our understanding of leadership roles and functions that are conducive to facilitating broad-scale change through individual and team learning in CoPs. For example, findings from this study shed light on the emergence of boundary-spanning roles within the cohort, an indication that CoP sponsors and nominated boundary-spanners could benefit from applying research-validated leadership models [47]. Further, an applied understanding of what helps and hinders the existence of CoPs in complex healthcare settings has the potential to mature our understanding of how CoPs influence complex adaptive systems change.

## Conclusion

In large, diverse and complex systems, no one person or group of people can have access to all the knowledge relevant to decision-making within the organisation at any given time. CoPs enable the diverse wealth of knowledge embedded in people, local conditions and special circumstances to flow from practice-specific domain groups to programme and service areas, and into the larger system where it can effect organisational change. In return, CoPs can use knowledge distilled from the broader organisation to advance their internal agendas such as improving practice and patient care, building effective information pathways, and engaging in team learning.

This leaves CoP members able to act on the information, specific needs and interests that motivate them, while leaving designated change agents and planners free to use CoP outputs for operational benefit. Examples of the latter include improved staff engagement and team learning, expedited change adoption, risk management, and the boundary-spanning function [42, 43].

The potential value and role of CoPs in organisational change was more visible at the macro or collective level of observation, and less visible at the micro or case-specific level. Using small-scale resource investments, the collective influence of CoP activities described in this paper advanced the study organisation’s progress along its integration journey involving complex system change. As a learning organisation practice, this study suggests that CoPs may have the potential to influence and advance widespread systems change in the Canadian healthcare system.

## Supplementary information

**Additional file 1.** Pre-screening criteria.

**Additional file 2.** Study definitions: structural and functional attributes of CoPs.

## Data Availability

Study data is not publicly available due to information that could compromise participant confidentiality and anonymity. The interview guide is available as a supplementary file on request of the authors.

## Abbreviations

AHS: Alberta Health Services
CoP: Community of Practice
CoPFN: Community of Practice Facilitators’ Network
RHA: Regional Health Authority
