# Supplementary material for: Communities of practice in Alberta Health Services: advancing a learning organisation
Source: Health Res Policy Syst. 2020 Aug 3;18:86. doi: 10.1186/s12961-020-00603-y (PMC7397570; doi:10.1186/s12961-020-00603-y)
Supplement: Supplementary file 1 — Additional file 1. Pre-screening criteria. [file 12961_2020_603_MOESM1_ESM.docx]

**Additional file 1**

Pre-screening criteria

| Peer leadership |
| --- |
| Voluntary participation |
| Regularly scheduled meetings |
| Membership inclusive of direct healthcare providers |
| A professional development focus with an aim to improve frontline patient care |
| Goals involving:   - - Supporting member well-being   - Facilitating information transfer   - Promoting communication   - Advancing practice insights   - Providing a problem-solving forum to help solve every-day work challenges   - Developing and disseminating best practices, guidelines and procedures   - Fostering collaboration and sharing across a geographically dispersed work force   - Fostering breakthrough ideas, knowledge, and practices |
