# Supplementary material for: Communities of practice in Alberta Health Services: advancing a learning organisation
Source: Health Res Policy Syst. 2020 Aug 3;18:86. doi: 10.1186/s12961-020-00603-y (PMC7397570; doi:10.1186/s12961-020-00603-y)
Supplement: Supplementary file 2 — Additional file 2. Study definitions: structural and functional attributes of CoPs. [file 12961_2020_603_MOESM2_ESM.docx]

**Additional File 2**

Study definitions: structural and functional attributes of CoPs

| CoP ATTRIBUTES  (structural-functional) | STUDY DEFINITIONS | BASIS OF CATEGORIES |
| --- | --- | --- |
| Practice domain | A shared area of membership expertise or interest | Broad areas of expertise within AHS and professional practice |
| Focus | Primary need/reason/purpose for coming together | Terminology commonly used by study participants |
| Membership boundaries | The extent to which membership is influenced, bordered and configured | A grouping of 4 attributes relating to practice domain, member roles, geographic boundaries and confidentiality restrictions |
| Meeting attendance | Average number of members attending meetings by range e.g. (50-100) | Estimates from memory by study participants |
| Meeting method | In-person and technology assisted means by which meetings are held | Terminology commonly used by study participants |
| Sphere of influence | The geographical and functional spheres of influence of a CoP | Focus and scope limited to a single geographic zone, spread over multiple geographic zones, organization-wide, or Alberta-wide |
